# New Insights into Chronic Pancreatitis: Potential Mechanisms Related to Probiotics

**DOI:** 10.3390/microorganisms12091760

**Published:** 2024-08-24

**Authors:** Yingyu Pan, Jianing Li, Zhengyang Fan, Yonghao Chen, Xiaoxuan Huang, Dong Wu

**Affiliations:** Department of Gastroenterology, State Key Laborotary of Complex Severe and Rare Diseases, Peking Union Medical College Hospital, Chinese Academy of Medical Sciences and Peking Union Medical College, Beijing 100730, China; panyy20@student.pumc.edu.cn (Y.P.); lijianing@263.net (J.L.); drfanzhengyang@126.com (Z.F.); chenyonghao2021@163.com (Y.C.); 202101015@student.pumc.edu.cn (X.H.)

**Keywords:** chronic pancreatitis, gut microbiota, prebiotics, probiotics

## Abstract

Chronic pancreatitis is a progressive fibroinflammatory disorder with no currently satisfactory treatment. Emerging evidence suggests an association between gut microbial dysbiosis and chronic pancreatitis. Although direct causative evidence is lacking, it is hypothesized that the gut microbiota may play a pivotal role in modulating pancreatic function via the gut–pancreas axis. Thus, modulating the gut microbiota through the administration of probiotics or prebiotics may alleviate pancreatic disorders. In this review, we first propose the potential mechanisms by which specific probiotics or prebiotics may ameliorate chronic pancreatitis, including the alleviation of small intestinal bacterial overgrowth (SIBO), the facilitation of short-chain fatty acids’ (SCFAs) production, and the activation of glucagon-like peptide-1 receptors (GLP-1Rs) in the pancreas. Since there are currently no probiotics or prebiotics used for the treatment of chronic pancreatitis, we discuss research in other disease models that have used probiotics or prebiotics to modulate pancreatic endocrine and exocrine functions and prevent pancreatic fibrosis. This provides indirect evidence for their potential application in the treatment of chronic pancreatitis. We anticipate that this research will stimulate further investigation into the gut–pancreas axis and the potential therapeutic value of probiotics and prebiotics in chronic pancreatitis.

## 1. Introduction

Chronic pancreatitis (CP) is a progressive fibroinflammatory syndrome with an annual incidence from five to eight and a prevalence of 42–73 cases per 100,000 adults in the United States [1,2,3]. With repetitive episodes of inflammation, the pancreas is irreversibly replaced by fibrotic tissues, resulting in chronic abdominal pain, endocrine and exocrine insufficiency, a reduced quality of life, and a shorter life expectancy [4]. Current therapeutic approaches primarily focus on symptom alleviation and supportive care, rather than targeting the underlying pathophysiological mechanisms [5].

**Figure 1:**
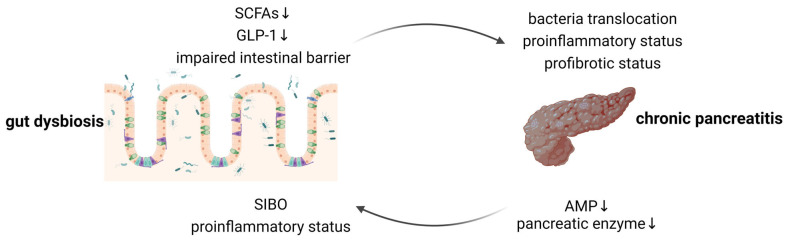
Bidirectional gut–pancreas interactions in the context of CP. The arrow indicates that one side has influence over the other. SCFA, short-chain fatty acid; GLP-1, glucagon-like peptide 1; SIBO, small intestinal bacterial overgrowth; and AMP, antimicrobial peptide. This figure was created with BioRender.com (accessed in July 2024).

In recent years, accumulating evidence has highlighted the crucial role of the pancreas in regulating the gut microbiota and the reciprocal influence of the gut microbiota on pancreatic function, which indicates the presence of a bidirectional relationship referred to as the “gut–pancreas axis”. The gut microbiota play a pivotal role in this axis through their involvement in metabolism and nutrition, protection against pathogens, and immune system regulation [6]. Bidirectional alteration of the gut–pancreas axis has been observed in many pancreatic diseases, including CP (Figure 1) [7]. Regarding its role in the homeostasis of the gut–pancreas axis, microbiota-based treatments, such as probiotics and prebiotics, may offer effective therapeutic options for CP.

Probiotics, defined as live microorganisms that confer health benefits to the host, and prebiotics, non-digestible food components that selectively stimulate beneficial gut bacteria, have shown potential in managing various gastrointestinal and systemic disorders [8]. In the context of CP, although probiotics or prebiotics have been proposed as potential treatments [6,7], their efficacy has not yet been validated in animal models or clinical trials. Additionally, their possible mechanisms of action have not been thoroughly explored. This study aims to explore the potential of probiotics and prebiotics to enhance pancreatic function and treat CP by investigating their effects on the gut–pancreas axis. We will discuss the possible mechanisms that ameliorate CP, including endocrine and exocrine function improvement, inflammation reduction, and pancreatic fibrosis alleviation. The effects of probiotics and prebiotics on these targets and their feasibility as intervention methods are reviewed.

It is important to emphasize that, to date, there have been no animal experiments or clinical trials specifically investigating the use of probiotics or prebiotics for treating CP. The mechanisms we propose here are derived from the clinical manifestations of CP, such as endocrine and exocrine insufficiency and pancreatic inflammation. We base our hypothesis on evidence from other disease models, where probiotics and prebiotics have shown potential in improving pancreatic function and alleviating symptoms. However, whether these mechanisms are applicable to CP remains an open question that future animal and clinical studies need to address. It must be acknowledged that much work remains to be conducted before probiotics, including engineered strains, and prebiotics can be considered for the clinical treatment of CP. We hope to inspire new approaches and provide fresh insights into the potential treatment of CP.

## 2. Search Strategy

**Figure 2:**
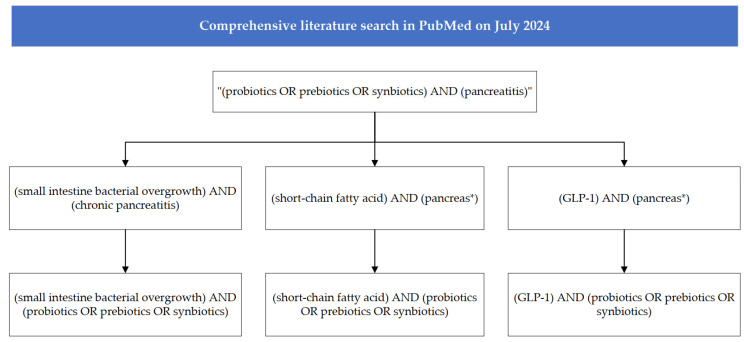
Flow chart of literature search for this review. Asterisks represent 0 to multiple characters (PubMed search tips).

We conducted a comprehensive search in PubMed in July 2024. The search strategy involved the following main queries: (1) “(probiotics OR prebiotics OR synbiotics) AND (pancreatitis)”. Literature and reference screening were conducted to select potentially relevant articles. This approach provided a general overview of the current research landscape and the potential therapeutic mechanisms of probiotics and prebiotics in CP. After identifying these potential mechanisms of action, the following search queries were included: (2) (small intestine bacterial overgrowth) AND (chronic pancreatitis); (3) (small intestine bacterial overgrowth) AND (probiotics OR prebiotics OR synbiotics); (4) (short-chain fatty acid) AND (pancreas*); (5) (short-chain fatty acid) AND (probiotics OR prebiotics OR synbiotics); (6) (GLP-1) AND (pancreas*); and (7) (GLP-1) AND (probiotics OR prebiotics OR synbiotics). The literature search process of this review is shown in Figure 2.

## 3. Alleviation of Small Intestinal Bacterial Overgrowth

In a healthy small intestine, several defective mechanisms maintain a relatively sterile environment: gastric acid secretion, an intact ileocecal valve, intestinal motility, immunoglobulins in intestinal secretions, and the bacteriostatic properties of pancreatic and biliary secretions [9,10]. When these protective mechanisms are disrupted, small intestinal bacterial overgrowth syndrome (SIBO) can occur. SIBO is characterized by an excessive number of bacteria in the small bowel, leading to gastrointestinal symptoms such as bloating, abdominal distension, diarrhea, and nutrient deficiencies [11,12]. A systematic review found that SIBO is present in 38% of patients with CP [13]. Current evidence links SIBO in CP to diabetes mellitus, pancreatic exocrine insufficiency, and the severity of CP, with treatment often resulting in symptomatic improvement [13,14,15,16].

The standard treatment for SIBO involves antibiotics aimed at eradicating the bacteria in the small intestine [17]. However, with a combined normalization rate of 51% for antibiotics, about half of patients may remain symptomatic despite treatment [18]. This necessitates refined treatment strategies. Probiotics and prebiotics are believed to benefit SIBO by preventing the growth of pathogenic flora through direct competition and the production of bacteriocins [12]. Several randomized controlled trials have shown that adding probiotics to antibiotic therapy results in higher clinical remission rates [19,20,21]. In a randomized prospective pilot study of patients with SIBO and chronic abdominal distension, the group receiving a combination of probiotics (*Lacticaseibacillus casei*, *Lactiplantibacillus plantarum*, *Streptococcus faecalis*, and *Bifidobacterium brevis*) showed significantly better clinical improvements compared to the sole metronidazole group [22]. A systematic review concluded that, while probiotics are unable to prevent SIBO, they can effectively decontaminate SIBO and relieve abdominal pain [23]. Probiotics also aid in repairing and reconstructing the intestinal mucosa. In rats treated with probiotic formulations containing coconut oil and traces of peppermint–lemon–patchouli essential oil, researchers observed mitotic figures and the regression of the inflammatory response in the villus epithelium and crypts previously damaged by SIBO-induced gut dysbiosis [24].

Probiotic supplementation to reduce SIBO has been attempted in various diseases, including irritable bowel syndrome [25,26,27,28,29], hypothyroidism during pregnancy [30,31,32], systemic sclerosis [33], liver diseases [34,35,36], and gastric and colorectal cancer [37]. However, there is a lack of research evidence on the application of probiotics for SIBO in chronic pancreatitis. Further investigation is needed to explore the potential benefits of probiotics in alleviating SIBO in CP.

## 4. Facilitation of Short-Chain Fatty Acids’ Production

Short-chain fatty acids (SCFAs), primarily acetate, propionate, and butyrate, are produced via the fermentation of dietary fibers by the gut microbiota. They have significant effects on various tissues, including the pancreas. Sodium butyrate is capable of inhibiting histone deacetylases (HDACs), which are crucial in inflammation and fibrogenesis. Post-treatment with sodium butyrate significantly reduces the expression of α-smooth muscle actin, interleukin-1β, inducible nitric oxide synthase, and 3-nitrotyrosine, thereby alleviating L-arginine-induced pancreatic damage and fibrosis in rats [38]. SCFAs modulate pancreatic fibrosis by inhibiting macrophage infiltration and M2 phenotype switching [39]. SCFAs have also been confirmed to play an immunoregulatory and anti-inflammatory role. Cathelicidin-related antimicrobial peptide (CRAMP) is an immunoregulatory antimicrobial peptide that can be produced by acinar cells. It modulates the phenotypic switching of intrapancreatic macrophages and changes the production of transforming growth factor-β, thereby defending against inflammation. Research has revealed that the production of CRAMP is regulated by SCFAs produced by the gut microbiota [40]. Additionally, SCFAs, especially butyrate, exhibit anti-inflammatory effects by inhibiting the activation of NF-κB and HDACs [41,42,43,44]. SCFAs also act directly on acinar cells to stimulate secretion, similar to incretins, through increasing the cellular calcium concentration [45,46,47,48].

Extensive studies have investigated SCFAs’ effects on insulin secretion, acting as ligands to G-protein-coupled receptors (GPCRs), specifically, free fatty acid receptor-2 (FFA2, previously termed GPR43) and FFA3 (previously termed GPR41). These receptors are found in various human tissues, including gut enteroendocrine cells and pancreatic islets [49,50]. FFA2 and FFA3 receptors on enteroendocrine cells trigger GLP-1 secretion [49], which has multiple positive effects and will be discussed in the next part. The enhanced secretion of insulin after SCFA treatment has been reported in a number of studies and is thought to be associated with FFA2 and FFA3 receptors on β-cells, but contradicting evidence also exists in several studies [50,51]. Therefore, no clear consensus has been reached on the effect of SCFAs on the FFA2 and FFA3 receptors in pancreatic islets.

Patients with CP exhibit a reduced abundance of SCFA producers, such as *Faecalibacterium* and *Fusicatenibacter* [52]. There was a noticeable reduction in *Faecalibacterium prausnitzii* in healthy controls compared to CP non-diabetics and CP diabetics [53]. The depletion of SCFA-producing Gram-positive bacteria in CP is independent of toll-like receptor 4 (TLR4), but supplementing exogenous SCFAs ameliorates the condition [39]. These studies implicated the role of SCFAs in protecting the pancreatic function from damage caused by CP. Therefore, supplementing probiotics or prebiotics that contribute to SCFA production may offer a novel intervention for managing CP.

Both in vivo and in vitro studies confirm that probiotics can increase SCFA levels. The probiotics capable of producing SCFAs are summarized in Table 1. In an in vitro human gut model, an aqueous probiotic suspension containing *L. plantarum*, *L. rhamnosus*, *L. acidophilus*, and *Enterococcus faecium* exerted anti-inflammatory effects through increased SCFA production, especially butyrate [54].

Prebiotics also show potential as clinical targets by promoting the growth and activity of probiotics. Prebiotics, typically complex carbohydrates such as starch, pectin, xylan, and arabinogalactan, serve as substrates for bacterial fermentation, resulting in the production of SCFAs [59]. The metabolism of different polysaccharides is associated with the production of different SCFAs. For example, pectin metabolism leads to a proportional increase in acetate concentration, while starch fermentation significantly boosts butyrate production over other SCFAs [60,61]. Overall, the microbial hydrolysis of insoluble substrates can promote the biosynthesis of high concentrations of SCFAs, with about 60% presenting as acetate, while butyrate and propionate each account for approximately 20% of gastrointestinal SCFAs [62]. Colonic SCFAs increase in healthy humans after consuming inulin or arabinoxylan-oligosaccharides-enriched food [63,64]. Inulin supplementation elevates the abundance of butyrate-producing microbiota, including *Bifidobacterium*, *Clostridium cluster* IV, and *Akkermansia muciniphila* [65]. When supplemented with oligofructose or inulin as the sole energy source, cross-feeding interactions between bifidobacteria and butyrate-producing bacteria like *Faecalibacterium prausnitzii* are observed. These interactions may enhance the colon ecosystem and contribute to combined bifidogenic and butyrogenic effects [66,67].

In summary, the use of probiotics and prebiotics to produce SCFAs shows promise as a management technique for CP. This approach could help to modulate inflammation, fibrosis, and pancreatic function, offering a potential therapeutic avenue worth further exploration.

## 5. Activation of Glucagon-like Peptide 1 Receptors in the Pancreas

Glucagon-like peptide 1 (GLP-1) is released from gut enteroendocrine cells at low levels during fasting and increases significantly within minutes of food digestion. GLP-1 is a multifaceted hormone with broad pharmacological potential, including incretin-like activity, the stimulation of glucose-dependent insulin secretion, and the inhibition of glucagon secretion, food intake, and gastric emptying [68,69]. Although GLP-1 is currently only approved for the treatment of T2DM and obesity [70], its multiple effects on both the endocrine and exocrine pancreas suggest the potential for alleviating symptoms of CP and slowing disease progression.

The physiological importance of GLP-1R in β-cells has been well-established in animal studies. GLP-1 normalizes glucose tolerance and enhances glucose-dependent insulin secretion via GLP-1R in pancreatic β-cells [71]. In addition to directly inhibiting glucagon secretion by α-cells [72], GLP-1 stimulates somatostatin secretion by δ-cells, further reducing glucagon levels [73,74]. Beyond regulating the blood glucose through modulating the levels of insulin and glucagon, GLP-1 inhibits β-cell apoptosis, induces β-cell proliferation, and increases β-cell mass [75]. In diabetic mouse models, GLP-1R activation alleviates endoplasmic reticulum stress in β-cells via the cAMP-dependent enhancement of activating transcription factor 4 (ATF4) translation, promoting β-cell survival [76]. Although GLP-1R agonists can increase the β-cell mass in diabetic rodent models, this effect is modest and short-lived, with older rodents showing reduced responses [77,78,79]. Nevertheless, these drugs are believed to help prevent further losses of β-cell mass and function, especially if treatment begins early in disease progression. In baboons subjected to partial pancreatectomy and treated with the GLP-1R agonist exenatide, immunofluorescent staining revealed ductal cells co-expressing insulin, suggesting that exenatide might promote the differentiation of ductal cells into β-like cells [80].

While most GLP-1 research focuses on α- and β-cells in the endocrine pancreas, GLP-1 also affects the exocrine pancreas. GLP-1R is expressed in a significant proportion of pancreatic acinar cells, though at lower levels than in β cells [81,82,83]. In caerulein-induced experimental pancreatitis, GLP-1R agonists increased the pancreas weight and induced anti-inflammatory protein expression while reducing proinflammatory markers [84]. Preclinical studies show that GLP-1R activation increases the acinar cell mass and protein content via S6 phosphorylation, independent of DNA content or cell proliferation changes [85]. GLP-1 has been shown to induce amylase secretion in the pancreatic acini in a dose-independent manner, potentially alleviating the weakened exocrine digestive capacity in CP patients [81,86]. Nevertheless, further investigation is still needed to exclude the potential harm of GLP-1 overactivation of the exocrine pancreas. A T2DM population-based cohort study reported a 1.5-fold increased risk of any pancreatitis and a 2.0-fold increased risk of AP among incretin users, with no increased risk for CP [87]. Chronic GLP-1R agonist treatment has been associated with the activation of pancreatic stellate cells (PSCs), which contributes to pancreatic fibrosis progression [88]. It remains to be explored whether there are ways to modify GLP-1R agonists to enhance their positive effects on pancreatic endocrine and exocrine functions while minimizing their impact on PSCs

In many animal models of other diseases, certain probiotics have been found to induce GLP-1 secretion (Table 2). In addition, using probiotics as oral vectors for recombinant GLP-1R agonist delivery has been explored to replace costly chemical synthesis and inconvenient injections. Probiotics can efficiently target the pancreas, offering a high bioavailability. *Lacticaseibacillus paracasei* L14 transformed with a plasmid encoding the exendin-4 gene showed efficient secretion and facilitated the transport of exendin-4, enhancing insulin secretion and maintaining β cells [89]. Engineered probiotic yeast *Saccharomyces boulardii* administered orally also produced bioactive GLP-1R agonists [90]. Apart from delivering GLP-1R agonists, protease-resistant modified GLP-1 (mGLP-1) was constructed with added arginine to ensure the structural integrity of mGLP-1 released in vivo [91]. In addition to producing bioactive GLP-1R agonists, engineered probiotics used as carriers also exert their inherent function of regulating the microbiota. Engineered *Clostridium butyricum* significantly improved gut microbiota dysbiosis in rats via downregulating the relative abundance of *Porphyromonadaceae* at the family level and upregulating *Lactobacillus* at the genus level [92]. Similarly, engineered *Escherichia coli Nissle* 1917 expressing GLP-1 regulated the intestinal flora and increased the probiotic diversity in mice [93].

An increase in GLP-1 secretion levels has also been observed following the addition of prebiotics, including dietary resistant starch [94,95], resistant maltodextrin [96], fructooligosaccharides [96,97], chondroitin sulfate [98], and Dendrobium officinale polysaccharide (DOP) [99]. The stimulative effect of prebiotics on GLP-1 secretion may occur through stimulating SCFA production [94,95].

**Table 2:** Summary of probiotics that can promote GLP-1 expression in various disease models.

Genus	Species	Disease Models	References
*Lacticaseibacillus*	*L. paracasei* L-21	STC-1 cell line	[100]
	*L. paracasei* JY062	Glycolipid metabolic disorders	[101]
	*L. casei* CCFM419	T2DM	[102]
	*L. rhamnosus* NCDC 17		[103]
*Lactiplantibacillus*	*L. plantarum subsp. plantarum* MTCC5690	T2DM	[104]
*Bifidobacterium*	*selenium-enriched B. longum* DD98	T2DM	[105]
	*B. animalis subsp. lactis* MN-Gup		[106]
	*B. animalis subsp. lactis NJ241*	Parkinson’s disease	[107]
	*B. animalis subsp. lactis* GCL2505	Metabolic syndrome	[108]
	*B. longum subsp. longum* B-53	STC-1 cell line	[100]
*Akkermansia*	Pasteurized *A. muciniphila*	T2DM	[109]
*Bacteroides*	*B. thetaiotaomicron*	alcoholic fatty liver disease	[110]
*Limosilactobacillus*	*L. fermentum* MG4295	T2DM	[111]
	*L. fermentum* MTCC5689		[104]
	*L. reuteri*	Glucose metabolism disorder induced by acrylamide; glucose-tolerant humans	[112,113]
*Clostridium*	*C. butyricum*	Chronic unpredictable mild stress; T2DM	[114,115]

In conclusion, the potential of engineered probiotics to express GLP-1 analogs offers a promising avenue for ameliorating symptoms in CP patients. Despite the current lack of experimental evidence regarding the use of engineered probiotics expressing GLP-1 in CP, their application holds significant promise considering their mechanisms of action and the positive effects observed in other disease models. This approach could improve pancreatic function and manage symptoms more effectively, but further research is needed to fully understand its implications and optimize treatment strategies.

## 6. Conclusions

Current treatments for CP lack innovation, underscoring the need for novel therapeutic approaches. The gut microbiota can influence pancreatic function through their metabolic activities in the gut, via the gut–pancreas axis. Probiotics and prebiotics may hold potential for treating CP via this axis.

The three possible intervention mechanisms discussed in this review—alleviating small intestine bacterial overgrowth, facilitating SCFAs’ production, and activating GLP-1R in the pancreas—are largely based on theoretical extrapolations from existing research, much of which is derived from other pancreatic disease models. Although there is a scarcity of experimental evidence specifically targeting CP, these mechanisms show strong potential for its treatment, including the improvement of pancreatic endocrine and exocrine functions and maintaining cellular and structural integrity. Therefore, there is an urgent need for experimental validation in the field of chronic pancreatitis. This exploration forms the core focus of this review, highlighting the promising potential of these interventions to address the pressing need for improved chronic pancreatitis therapies.

## Figures and Tables

**Table 1:** Main examples of probiotics producing short-chain fatty acids.

Probiotics	Products	References
*Bifidobacterium bifidum* H3-R2	Acetate	[55]
*Lactiplantibacillus plantarum* NC8	Acetate	[56]
*Lacticaseibacillus rhamnosus* HN001	Propionate	[57]
*Propionibacterium freudenreichii* B1	Propionate	[55]
*Clostridium butyricum* C1–6	Butyrate	[55]
*Lactobacillus acidophilus* KLDS 1.0901	Acetate, propionate, butyrate	[58]
*Lactiplantibacillus plantarum* KLDS 1.0386		

## Data Availability

The data underlying this article are available in the article.

## Abbreviations

CP: chronic pancreatitis; SIBO: small intestinal bacterial overgrowth; SCFA: short-chain fatty acid; GLP-1: glucagon-like peptide-1; GLP-1R: glucagon-like peptide-1 receptor; HDAC: histone deacetylase; CRAMP: cathelicidin-related antimicrobial peptide; NF-κB: nuclear factor kappa-B; GPCR: G-protein-coupled receptor; FFA2: free fatty acid receptor-2; FFA3: free fatty acid receptor-2; T2DM: type 2 diabetes mellitus; cAMP: cyclic adenosine monophosphate; PSC: pancreatic stellate cell.
